# Visual Personal Familiarity in Amnestic Mild Cognitive Impairment

**DOI:** 10.1371/journal.pone.0020030

**Published:** 2011-05-20

**Authors:** Luisa Jurjanz, Markus Donix, Eva C. Amanatidis, Shirin Meyer, Katrin Poettrich, Thomas Huebner, Damaris Baeumler, Michael N. Smolka, Vjera A. Holthoff

**Affiliations:** 1 Division of Old Age Psychiatry, Department of Psychiatry and Psychotherapy, University Hospital Carl Gustav Carus, Technische Universität Dresden, Dresden, Germany; 2 German Center for Neurodegenerative Diseases, Dresden, Germany; 3 Department of Psychology, Neuroimaging Center, Technische Universität Dresden, Dresden, Germany; Cuban Neuroscience Center, Cuba

## Abstract

**Background:**

Patients with amnestic mild cognitive impairment are at high risk for developing Alzheimer's disease. Besides episodic memory dysfunction they show deficits in accessing contextual knowledge that further specifies a general concept or helps to identify an object or a person.

**Methodology/Principal Findings:**

Using functional magnetic resonance imaging, we investigated the neural networks associated with the perception of personal familiar faces and places in patients with amnestic mild cognitive impairment and healthy control subjects. Irrespective of stimulus type, patients compared to control subjects showed lower activity in right prefrontal brain regions when perceiving personally familiar versus unfamiliar faces and places. Both groups did not show different neural activity when perceiving faces or places irrespective of familiarity.

**Conclusions/Significance:**

Our data highlight changes in a frontal cortical network associated with knowledge-based personal familiarity among patients with amnestic mild cognitive impairment. These changes could contribute to deficits in social cognition and may reduce the patients' ability to transition from basic to complex situations and tasks.

## Introduction

Familiarity describes the initial ‘feeling of knowing’ that immediately arises at the moment we recognize someone or something we were previously exposed to. For example, unexpectedly encountering a person we already met before, could elicit a feeling of familiarity although we may not be able to remember any specific details about this person [1]. This type of familiarity is based on repeated perception. In contrast, *personal* familiarity implies the availability of contextual knowledge which would individuate a stimulus [2]. In this case, recognizing an object or a person elicits multifaceted information, such as semantic knowledge (e.g., where a person lives or works, or what the person's intentions and feelings are), our emotional response towards the stimulus, or autobiographical episodes that come to our mind. The ability to not just recognize someone or something as already known, but to identify a stimulus as personally familiar based on contextual information and emotional response, is essential for our everyday functioning.

The potential influence of personal familiarity on object use and person recognition plays a substantial role in treatment and care of patients with pathological cognitive decline. For example, Giovannetti et al. [3] showed that patients with dementia performed significantly better in identifying personal objects versus unfamiliar analogs, or generating specific information and gestures for them. A familiar environment reduces wandering behavior [4], and patients may show impaired functional task performance in an unfamiliar environment, but the same skills could be preserved in a familiar environment [5]. Most importantly, the close relationship to a familiar person is associated with improved psychological wellbeing and better problem solving abilities [6], as well as slower cognitive decline [7].

In this study we investigated patients with amnestic mild cognitive impairment (aMCI). Although these patients are not demented, they are at high risk for developing Alzheimer's Disease (AD), with annual conversion rates of 10–12% [8]. According to the diagnostic criteria, aMCI patients present with memory complaint, which is preferably corroborated by an informant, objective memory deficits beyond what is expected for their age, relatively preserved general cognition, and intact activities of daily living [9]. Studies investigating familiarity in aMCI patients are rare and focus on experimentally learned (perceptual) familiarity using visual and auditory stimuli. This perceptual familiarity appears to be unimpaired among aMCI patients [10], [11], although this may depend on how familiarity is investigated [1]. There is no study specifically investigating *personal* familiarity in aMCI patients. Recent behavioral data suggest that aMCI patients may have difficulties in accessing episodic memory details [12], and in forming associations between different types of information [13]. As outlined above, both processes are essential for experiencing personal familiarity.

Studies aimed at investigating the neural correlates underlying familiarity are rare, and they usually focus on young, healthy people. In functional magnetic resonance imaging (fMRI) studies, familiar stimuli have been shown to activate medial posterior brain regions across various stimulus modalities, such as faces, places or voices [14], [15], [16], [17]. This suggests the existence of a unique brain network involved in familiarity perception, which is relatively independent from the stimulus modality [14], [18]. Activity in the posterior cingulate cortex and the precuneus can be demonstrated when we perceive experimentally learned stimuli [2], [15], [19], so it does not depend on the availability of background knowledge surrounding a stimulus. Studies specifically investigating the neural networks associated with *personal* (knowledge-based) familiarity revealed additional activity in medial prefrontal, anterior cingulate and posterior temporal areas [2], [20]. These areas are known to be associated with social cognition [21], the representation of the mental states of others [22], and self-referential processing [23]. The activity pattern may also reflect the richness of available episodic and semantic information associated with personal familiarity, as well as social attachment and emotional response [18], [20].

Recent structural and functional imaging studies investigating MCI patients suggest complex anatomical and functional changes in brain regions that are associated with familiarity [24], [25], [26], [27]. There is, for example, an increased beta amyloid deposition in the prefrontal cortex [24], [28], indicating that neural changes are not restricted to the medial temporal lobe, where earliest AD-associated neuropathology is known to arise [29]. In a recent positron emission tomography (PET) study, Fouquet et al. [30] demonstrated significant reduction in medial prefrontal and anterior cingulate glucose metabolism among aMCI subjects later converting to AD. The posterior cingulate cortex is also one of the brain regions showing hypometabolism in MCI [31], [32]. However, using fMRI, Ries et al. [33] demonstrated that healthy control subjects and aMCI patients showed similar posterior cingulate activity when they perceived self-associated information rather than experimentally learned information. This suggests that although metabolic changes in the posterior cingulate cortex may occur early in the course of pathologic cognitive decline, this does not necessarily reflect functional impairment in a specific situation.

To reveal the neural networks associated with personal familiarity, in this study, photographs of personally familiar faces (spouse or children) and places (from the participants' own homes), as well as unfamiliar faces and places, were presented to aMCI patients and healthy elderly control participants during fMRI scanning. We predicted that in line with the ability to recognize the visual stimulus as familiar, both participant groups would engage the posterior cingulate/precuneus region irrespective of stimulus type (face/place), when perceiving familiar versus novel context. We further hypothesized that associated with impairment in accessing rich contextual details for the familiar stimulus, aMCI patients would exhibit reduced activity in prefrontal cortical areas.

## Methods

### Subjects

All aMCI subjects were recruited through the University's Memory Clinic. Control participants responded to public advertisements. The experiments were done in accord with the Helsinki Declaration of 1975. The Ethics Committee of Dresden University's Medical Faculty ‘Carl Gustav Carus’, Dresden, Germany approved the study and written informed consent was obtained. Twelve aMCI subjects meeting Petersen et al. [9] criteria participated in the study. All aMCI subjects had subjective memory complaints, were not demented and reported normal activities of daily living. The aMCI patients showed memory impairment, which was defined as a performance of one standard deviation below age-adjusted normative data in at least one of the tests of verbal or non-verbal long-term memory (CVLT [34], WMS-R [35]) at the single subject level. Performance in other cognitive domains was within the age-adjusted normal range (immediate recall or working memory: WMS-R [35]; language: AAT, COWAT [36], [37], processing speed and attention: Trailmaking Test A and B [38]. Diagnoses were established by a clinician after clinical patient evaluation and the neuropsychological examination shown in **Table 1**. Standard laboratory testing and structural brain scans complemented the diagnostic procedures to rule out conditions that would have explained the cognitive impairment otherwise. All aMCI subjects were classified as single-domain type [9].

**Table 1:** Demographic and neurocognitive characteristics.

Characteristic	Controls (n = 12)	aMCI (n = 12)		t-Test, 2-tailed	
	Mean (SD)	Mean (SD)	d^2^	t_(22)_	p
age	62.1 (5.4)	66.6 (8.7)	0.08	−1.52	0.14
female sex (no.)	6	6			
Education (years)	11.1 (1.4)	10.8 (1.3)	0.16	0.61	0.55
BDI	4.50 (4.4)	6.20 (4.4)	0.04	−0.91	0.38
MMSE (raw)	29.58 (0.52)	28.00 (1.81)	1.19	2.92	**0.01**
*Verbal memory*					
CVLT, List A^1^	0.92 (1.24)	−0.42 (1.24)	1.08	2.63	**0.02**
CVLT, List B	−0.58 (1.31)	−1.08 (0.52)	0.50	1.23	0.24
CVLT, short delay free recall	0.42 (1.08)	−0.92 (1.24)	1.15	2.81	**0.01**
CVLT, short delay cued recall	0.33 (0.65)	−0.58 (1.31)	0.88	2.17	**0.04**
CVLT, long delay free recall	0.33 (1.23)	−0.83 (1.27)	0.93	2.29	**0.03**
CVLT, long delay cued recall	0.50 (0.52)	−0.67 (1.44)	1.08	2.65	**0.02**
CVLT, recognition hits	0.08 (0.67)	−0.42 (1.17)	0.52	1.29	0.21
*Visual memory*					
WMS-R, visual memory immediate recall	1.42 (0.76)	0.60 (1.25)	0.79	1.93	0.07
WMS-R, visual memory delay	1.37 (0.83)	0.22 (1.62)	0.89	2.20	**0.04**
*Working Memory*					
WMS-R, digit span forward	1.04 (0.67)	0.70 (1.10)	0.36	0.91	0.38
WMS-R, digit span backward	0.90 (0.99)	0.52 (1.02)	0.37	0.94	0.36
*Language*					
FAS: F	0.96 (1.06)	0.56 (1.00)	0.41	0.95	0.35
FAS: A	1.38 (1.96)	1.59 (0.81)	0.14	−0.35	0.73
FAS: S	0.58 (0.94)	0.27 (0.50)	0.23	1.01	0.32
AAT, pictured objects (single nouns)	0.82 (0.00)	0.68 (0.46)	0.29	1.00	0.33
AAT, pictured objects (compound nouns)	0.71 (0.45)	0.54 (1.05)	0.21	0.53	0.61
*Attention*					
WMS-R, mental control	0.40 (0.55)	0.58 (0.48)	0.35	−0.85	0.41
Trailmaking test A (raw)	37.1 (13.95)	42.5 (14.28)	0.38	−0.94	0.36
Trailmaking test B (raw)	84.8 (48.67)	115.8 (34.94)	0.73	−1.79	0.09

1 age-adjusted z-values unless otherwise indicated.

2 effect size measure (Cohen's d).

BDI: Beck Depression Inventory; MMSE: Mini Mental State Examination; CVLT: California Verbal Learning Test; WMS-R: Wechsler Memory Scale – Revised; FAS: Controlled Oral Word Association Test, letters F,A,S; AAT: Aachen Aphasia Test.

Twelve cognitively healthy subjects participated as control subjects. These subjects performed within the normal range in all neuropsychological tests. The healthy subjects also served as participants in a previous study investigating familiarity effects in normal aging [39]. In both groups, only subjects free of white matter lesions or focal white matter lesions only (ARWMC-scale [40], score<2 points) and free of focal lesions in grey matter were included. All subjects were right-handed. Exclusion criteria were education less than eight years, history of alcohol or substance abuse, head trauma, psychiatric or neurological disorder preceding MCI onset, or major systemic disease affecting brain function. All study participants were free of any medication aimed at stabilizing or enhancing cognitive functioning.

### Image preparation

For familiar faces, we obtained photographs of each participant's close relatives (spouse or children) with a digital camera. Each relative was photographed from five different angles (left side; 45° left, frontal, 45° right, right side). Images were digitally manipulated to ensure similar head size, luminance, and background. Pictures of unfamiliar faces were obtained from family members of the clinical staff. Familiar and unfamiliar face stimuli were matched for gender and approximate age. Images of familiar places were taken of the participants' homes. We obtained photographs of rooms rather than of single furniture. Pictures of unfamiliar places were obtained from the homes of clinical staff members and their relatives.

### Experimental design

In order to investigate the neural activity associated with different stimulus modality and personal familiarity, we used a blocked factorial design, presenting images of personally familiar faces and places, and unfamiliar faces and places during an fMRI experiment. We utilized the same experimental procedure as in our previous study investigating familiarity effects in healthy aging [39]. Briefly, five individual stimuli of one of the four conditions (familiar face = FF, unfamiliar face = UF, familiar place = FP, unfamiliar place = UP) were blocked together (stimulus onset-time 5s). Each block's image showed the same stimulus but photographed from different angles to avoid habituation effects. Images were presented in counterbalanced order within and between subjects for both familiarity and stimulus modality. To ensure alertness, and to test whether participants would correctly recognize familiar and unfamiliar stimuli each block contained a question stimulus in response of which the subjects were asked to press the correct button (“if the stimulus presented was familiar press the button in your left hand/if unfamiliar press the button in your right hand”). Experimental conditions were separated by intervals lasting 9s, during which the participants focused at a fixation cross. We performed a total of three experimental runs, each consisting of 8 stimulus blocks. Given this design, each condition was presented six times (twice per run) in the experiment. Across these six presentations, stimulus images were not repeated; therefore the participant did not see the same image twice throughout the experiment. We used a 3T MRI scanner (Trio; Siemens AG, Erlangen, Germany). fMRI images were acquired with an EPI pulse sequence using BOLD contrast: TR = 1.95 s, TE = 25 ms, α = 80°, 34 transversal slices acquired in descending order, orientated axially parallel to the ac-pc line, thickness 3 mm (1 mm gap), FOV = 220 mm, voxel size 3.44×3.44×4 mm. We collected 547 volumes for each subject. Stimuli were presented using bi-screen goggles, placed next to the subject's eyes below the head coil (VisuaStim Digital, Resonance Technology Inc., Northridge, CA, USA). Task presentation and behavioral response recording was performed with Presentation® software (Version 9.9, Neurobehavioral Systems Inc., Albany, CA, USA). High-resolution anatomic images were also acquired using a T1-weighted 3-D magnetization-prepared, rapid acquisition gradient echo (MPRAGE) pulse sequence: TR = 1.9 s, TE = 2.26 ms, FOV = 256 mm, 176 slices, voxel size 1×1×1 mm^3^.

### Image processing and statistical analysis

Image processing and statistical calculations were performed using MATLAB (The Mathworks Inc., Natick MA, USA) and statistical parametric mapping software (SPM5, Wellcome Department of Imaging Neuroscience, London, UK). The first five EPI images were discarded to allow the MRI signal to reach a steady state. To correct for head movement we spatially realigned individual data to the first volume. We used a standard EPI template (MNI brain) for normalization. After resampling to achieve 3×3×3 mm isotropic voxels we smoothed the functional data using an isotropic Gaussian kernel of 10 mm FWHM. At the single subject level, we modelled all four conditions of the paradigm in the context of a general linear model (GLM). We also modelled the question stimulus, the subjects' response (button presses) and feedback separately from the rest condition (focusing on a fixation cross). We used a flexible factorial modelling procedure for second level analyses in a 2*2*2 factorial design, investigating the factors stimulus type (face/place), familiarity (familiar/unfamiliar), and group (control/aMCI). After examining the factors' main effects, we investigated all two-way interactions (group*familiarity, group*stimulus type and familiarity*stimulus type). In case of significant interactions we additionally calculated the respective simple main effects (e.g. effect of familiarity in both groups). Although our groups did not differ in mean age or education status, we additionally investigated whether modelling age and education as covariates would change our findings. Voxels in MNI-space were considered statistically significant at a threshold of p<0.05 (corrected at cluster level) using a height threshold of p<0.001 uncorrected, corresponding to T = 3.28 and a cluster size of at least 30 activated voxels. Sociodemographic data and neuropsychological scores were compared using two-tailed t-tests.

## Results

### fMRI

**Figure 1:**
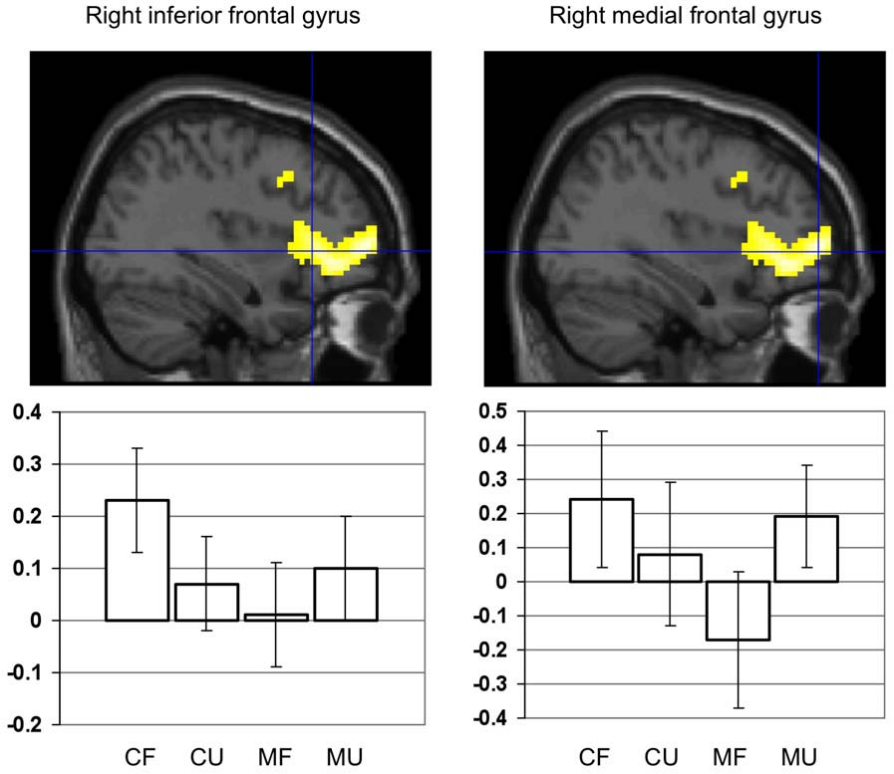
Between-group comparison: personal familiarity irrespective of stimulus type. The figure shows brain areas with relative decrease in neural activity among aMCI patients when compared to control subjects, associated with familiar>unfamiliar stimulus content irrespective of stimulus type. The two local maxima (indicated by crosshair positions) are superimposed on a sagittal single subject brain section provided by SPM5. Both maxima are part of the same cluster (for details see Table 3). The histograms display percentage BOLD signal change for the local maximum as a function of the experimental conditions (mean and 90% confidence interval). CF = controls familiar, CU = controls unfamiliar, MF = aMCI familiar, MU = aMCI unfamiliar.

**Figure 2:**
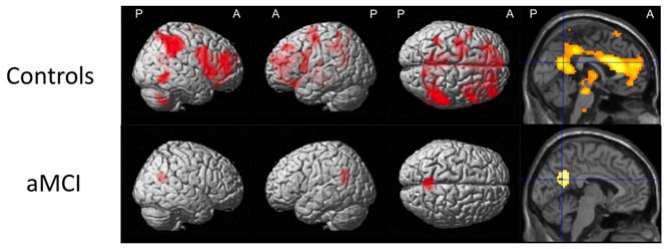
Within-group effect of personal familiarity irrespective of stimulus type. The figure shows brain areas with relative increase in neural activity for both subject groups when perceiving familiar>unfamiliar stimulus content irrespective of stimulus type. The local maxima are superimposed on a rendered standard single subject brain provided by SPM5. See Table 3 for exact coordinates. R = right, L = left, A = anterior, P = posterior.

Given our hypotheses, we were specifically interested in examining a possible interaction between the factors group and familiarity. We detected significant main effects for familiarity but not for group (**Table 2**), as well as a significant interaction between both factors. Group comparison (interaction group*familiarity) revealed lower right prefrontal cortical activity among aMCI subjects when compared to control participants for familiar versus unfamiliar stimuli (Contrast (FF+FP)-(UF+UP), **Table 3** **,** **Figure 1**). Group comparison for the reverse effect (controls<aMCI) did not yield a significant finding. To further explore the different patterns of brain activation between controls and aMCI patients we calculated the effect of familiarity within both groups (simple main effects familiarity). Among control subjects, familiar compared to unfamiliar stimuli, irrespective of stimulus type (contrast (FF+FP)-(UF+UP)), elicited substantially more brain activity, primarily in frontal, anterior and posterior cingulate, as well as temporal areas. aMCI subjects showed a bilateral activation in the precuneus for this contrast, extending into the right posterior cingulate cortex (**Table 3** **,** **Figure 2**). In both groups, there was no brain region showing less activity associated with familiar compared to unfamiliar stimuli.

**Table 2:** fMRI: factor main effects.

Region	Side	x	y	z	T-Score	k_E_ (voxels)
**Main effect of familiarity: familiar>unfamiliar**						
Precuneus	L	−9	−54	30	7.76	3840
Anterior cingulate	L	−9	45	15	7.69	#
Inferior parietal lobule	R	57	−36	48	6.90	#
Right precuneus	R	6	−60	21	6.41	#
Anterior cingulate	L	−3	30	15	5.95	#
Anterior cingulate	R	6	30	15	5.90	#
Inferior frontal gyrus	R	48	6	18	5.93	325
Cerebellum	R	60	−57	−9	5.09	195
Inferior temporal gyrus	R	18	−60	−51	4.91	197
Postcentral gyrus	L	−39	−45	63	4.83	439
**Main effect of familiarity: unfamiliar>familiar**						
Postcentral gyrus	R	39	−21	51	5.85	413
Precentral gyrus	R	39	−24	60	6.31	#
Rolandic operculum	R	45	−24	21	5.23	218
**Main effect of group: control>aMCI**-no suprathreshold clusters						
**Main effect of group: aMCI>control**-no suprathreshold clusters						
**Main effect of stimulus type: faces>places**						
Middle temporal gyrus	R	57	−63	9	9.97	1087
Supramarginal gyrus	R	54	−27	24	3.77	#
Angular gyrus	R	57	−57	36	3.69	#
Middle temporal gyrus	L	−54	−57	12	6.15	569
Precuneus		0	−63	33	5.00	136
Temporal pole	R	30	9	−24	4.63	130
**Main effect of stimulus type: places>faces**						
Fusiform gyrus	L	−27	−45	−15	19.66	9651
Middle occipital gyrus	L	−36	−87	21	17.46	#
Middle occipital gyrus	R	36	84	21	17.22	#
Fusiform gyrus	R	30	−39	−15	16.49	#
Lingual gyrus	R	18	−75	−6	14.20	#
Precentral gyrus	R	30	−3	51	5.10	159
Superior frontal gyrus	R	30	15	63	3.72	#

All activations are significant at p<0.05, corrected for multiple comparisons at the cluster level (with a height threshold of p<0.001, uncorrected at the voxel level). For each region of activation, the coordinates of the maximally activated voxels within the activation cluster are given in standard stereotactic MNI space. # indicates that this activation maximum is part of the same cluster.

**Table 3:** Relative increases in brain activity associated with personal familiarity.

Region	Side	x	y	z	T-Score	k_E_ (voxels)
Simple main effect familiarity: (FF+FP)-(UF+UP)						
**Control subjects**						
Anterior cingulate	L	−9	45	17	7.11	5553#
Posterior cingulate	L	−12	−51	30	6.67	#
Middle frontal gyrus	L	−24	30	39	4.67	102
Inferior frontal gyrus	L	−30	24	−18	5.07	133
Inferior frontal gyrus	L	−51	6	12	4.62	174
Precentral gyrus	L	−33	−12	51	4.62	219
Inferior parietal lobule	L	−36	−42	45	4.25	108
Cerebellum	R	33	−54	−51	5.58	130
Inferior temporal gyrus	R	51	−51	3	5.17	151
**aMCI patients**						
Precuneus	L	−6	−61	35	4.29	174
	R	6	−61	29	3.68	#
Group comparison (interaction group*familiarity)						
**Controls>aMCI**						
Inferior frontal gyrus	R	45	20	8	4.90	709
Medial frontal gyrus	R	39	53	8	4.89	#

All activations are significant at p<0.05, corrected for multiple comparisons at the cluster level (with a height threshold of p<0.001, uncorrected at the voxel level). For each region of activation, the coordinates of the maximally activated voxels within the activation cluster are given in standard stereotactic MNI space. FF: familiar faces, UF: unfamiliar faces, FP: familiar places, UP: unfamiliar places; # indicates that this activation maximum is part of the same cluster.

The factor stimulus type was of no primary interest for our main hypothesis. However, investigating main effects (**Table 2**) for stimulus type (irrespective of familiarity, contrast (FF+UF)−(FP+UP)), we found that subjects showed more brain activity in bilateral temporal areas when perceiving faces compared to places. Contrariwise presentation of places elicited more brain activity in occipital brain regions. Investigating the interaction terms stimulus type*group and stimulus type*familiarity did not reveal brain regions showing significantly greater or reduced neural activity associated with one of the conditions. Modelling age and education as covariates did not change the pattern of our significant group findings.

### Post-scanning debriefing

In a post-scanning debriefing, individual stimuli used during the scan were again presented on a computer screen. Both participant groups did not significantly differ in their ability to correctly categorize familiar and unfamiliar stimuli.

## Discussion

In this study we demonstrated that aMCI patients compared to healthy elderly subjects showed lower activity in right prefrontal brain regions when perceiving personally familiar faces and places. Within-group comparison revealed that control participants activated a large neural network including frontal, posterior cingulate and temporal cortices for personally familiar versus unfamiliar stimuli, whereas aMCI patients showed activity in the bilateral precuneus and right posterior cingulate cortex only. These differences in neural activity occurred irrespective of visual stimulus modality (face/place), and despite the fact that both groups did not show neural activity differences when perceiving faces or places per se, irrespective of familiarity.

Personal familiarity associated with close family members and one's own home arises from years of interaction and exposure. The recollection of specific knowledge and experiences associated with a familiar stimulus has been shown to recruit brain regions involved in social cognition and episodic memory [2], [21], [41]. For example, Cloutier at al. [2] demonstrated that the extensive information surrounding a familiar face stimulus is preferentially associated with neural activity in medial prefrontal cortex. Within this region, the anterior cingulate and paracingulate cortices, and the anterior frontal poles play a major role in episodic memory retrieval [41], self-reflection [23], and making inferences about others' thoughts [42].

Throughout the literature there is a main focus on the medial temporal lobe with respect to patients suffering from pathologic cognitive decline. Our data contribute to the emerging evidence that changes in frontal cortical functioning are also involved relatively early in the course of cognitive impairment. Due to its late myelination in brain development, the frontal cortex is susceptible to myelin damage [43]. Elevated beta amyloid levels in the prefrontal cortex of aMCI patients [24] could contribute to myelin toxicity [44]. AD patients show functional disconnection, particularly between medial temporal and frontal cortical areas [45], [46]. This model of disease pathophysiology is supported by brain metabolic changes, such as reduced frontal cortical glucose metabolism among aMCI subjects later converting to AD [30], [47]. Our fMRI results indicate reduced prefrontal cortical activity associated with a personally familiar stimulus in aMCI patients. Previous data show that aMCI patients are impaired in accessing specific memory details and forming associations between different types of information [12], [13]. The lower frontal brain activity may reflect impairment in connecting rich background information with a familiar stimulus. Although this will require future investigations, our data are in line with the notion that cognitive impairment in aMCI patients is more complex than episodic memory retrieval deficit detected by standard neuropsychological testing. Reduced frontal cortical activity could reflect subtle changes in working-memory capacity [13] and executive functioning [48]. However, it needs to be mentioned that there are conflicting data whether cognitively impaired patients at risk for AD would show increased [49], [50] or decreased [51], [52] frontal cortical activity during memory tasks. These differences could reflect different stages of cognitive impairment [53], or they could be task-associated [54].

In contrast to prefrontal cortical activity, we did not detect a group difference in the posterior cingulate cortex when the subjects perceived personally familiar stimuli. The posterior cingulate/precuneus region is closely associated with perceptual familiarity, irrespective of whether or not there is any knowledge available that would further individuate the perceptually familiar stimulus [2], [15], [19]. A number of studies show preserved perceptual familiarity recognition in aMCI [10], [11], [55], contrary to the patients' declining memory recollection abilities. The brain network involved in familiarity recognition seems to be relatively independent from the stimulus modality [15], [16], [17], [18]. It is therefore interesting that a *personally* familiar environment is particularly helpful in dementia care and therapy [3], [5], [7], since a demented person may not have access to semantic facts or episodic memories surrounding a familiar face or object. Besides the availability of such contextual knowledge, this could also be due to the emotional salience of a stimulus, which is known to influence familiarity-associated neural activity in the posterior cingulate cortex [20], [56]. Other investigations demonstrated that the degree of self-relevance of a familiar stimulus may also modulate the activity in the posterior cingulate/precuneus region [33], [57].

With respect to activation laterality, it should be highlighted that our group difference associated with personal familiarity was detected in the right prefrontal cortex. Whereas left more than right prefrontal regions are involved in episodic memory encoding, the opposite pattern has been described for episodic memory retrieval [58]. Thus, our data could suggest an early impairment in accessing semantic and episodic information associated with a familiar stimulus among aMCI patients, which would be in line with the existing literature [12], [59]. However, this has to be interpreted with caution. Aging itself could influence hemispheric lateralization processes [60] and frontal cortical involvement in general [61]. We previously demonstrated that frontal cortical activity associated with perceiving a personally familiar face or place, did not change with age [39]. In this study we would therefore not expect familiarity-related hemispheric lateralization effects to be aging-associated. It remains a possibility that right prefrontal cortices may be preferentially involved in encoding pictorial rather than verbal information [62], which could have contributed to a lateralization effect.

It is a limitation of this study that we were not able to directly investigate the participants' performance of retrieving detail-rich contextual information on the behavioral level. However, we previously showed that aMCI patients retrieve autobiographical events with fewer details when compared to healthy subjects [12]. Finally our data may be susceptible to false negative findings due to the small sample size. We reanalyzed our data using a height threshold of p<0.05, uncorrected. This did not change the pattern of brain regions for which we found a significant group difference.
